# Reproductive Power of Filth

**Published:** 1856-09

**Authors:** 


### REPRODUCTIVE POWER OF FILTH.

A single atom of Spanish moss attaches itself to a southern tree, every moment and hour, day and night, summer and winter, it steadily extends itself, until the whole tree is hung in the drapery of death.

The toad-stool mushroon, so deadly in its nature, is the work of a night, and augments with wonderful rapidity.

So it is with a low grade of animal and vegetable growth, which feeds on filth, and reproduces itself with the utmost celerity, thus spreading its area, and concentrating its corrupting and destructive agencies, sweeping away human life like chaff.

These pernicious growths, scarcely themselves perceptible to the naked eye, have something immeasurably more minute, which answer to seeds, which flying in every direction, and attaching themselves to all moist surfaces, begin instantly to grow. Thus it is, that spots of neglected filth need but a little moisture and warmth to breed their deadly contagions, and scatter their leprous diseases far and wide.

Let every family, then, remember that each particle of damp dirt about their dwellings is a plague spot, and let every servant and child be visited with the severest reproof, who knowingly permits its continuance for a single moment.

